# Increased sensitivity of NS1 ELISA by heat dissociation in acute dengue 4 cases

**DOI:** 10.1186/s12879-017-2306-z

**Published:** 2017-03-11

**Authors:** Sibelle Nogueira Buonora, Flavia Barreto dos Santos, Regina Paiva Daumas, Sonia Regina Lambert Passos, Manoela Heringer da Silva, Monique Rocha de Lima, Rita Maria Ribeiro Nogueira

**Affiliations:** 1 National Institute of Infectious Diseases Evandro Chagas, Laboratory of Clinical Epidemiology, Oswaldo Cruz Foundation, Av. Brasil, 4036 sala 201 A – Manguinhos, Rio de Janeiro, CEP: 21040-361 Brazil; 2 Oswaldo Cruz Institute, Instituto Oswaldo Cruz – Pavilhão Hélio e Peggy Pereira, Flavivirus Laboratory, Oswaldo Cruz Foundation, Avenida Brasil, 4365, Manguinhos, Rio de Janeiro, CEP: 21040-900 Brazil; 3 Germano Sinval Faria Teaching Primary Care Center, National School of Public Health, Oswaldo Cruz Foundation, Rua Leopoldo Bulhões, 1480 - Bonsucesso, Rio de Janeiro, CEP: 21041-210 Brazil

**Keywords:** NS1 ELISA, Accuracy, Dengue diagnosis, Immune complex dissociation, DENV-4

## Abstract

**Background:**

Dengue is an acute febrile illness considered the major arboviral disease in terms of morbidity, mortality, economic impact and dissemination worldwide. Brazil accounts for the highest notification rate, with circulation of all four dengue serotypes. The NS1 antigen is a dengue highly conserved specific soluble glycoprotein essential for viral replication and viability that can be detected 0 to 18 days from the onset of fever (peak first 3 days). It induces a strong humoral response and is known as a complement-fixing antigen. Lower NS1 test sensitivity occurs in secondary dengue infections probably due to immune complex formation impairing antigen detection by ELISA.

**Methods:**

We compared the sensitivity of NS1 ELISA in heat dissociated and non-dissociated samples from 156 RT-PCR confirmed acute dengue-4 cases from 362 prospectively enrolled patients.

**Results:**

Secondary infections accounted for 83.3% of cases. NS1 ELISA was positive in 42.5% and indeterminate in 10.2% of dengue-4 cases. After heat dissociation, 7 negative and 16 indeterminate samples turned positive, increasing the overall test sensitivity to 57.7%.

**Conclusions:**

Although it is time consuming and requires the use of specific laboratory equipment, NS1 ELISA combined with heat dissociation could be a slightly better alternative for triage in suspected dengue cases.

## Background

Dengue is an acute febrile illness considered the major worldwide arboviral disease, in terms of morbidity, mortality, economic impact and dissemination [1]. Dengue occurs in all non-polar continents [2] and is an important public health concern in tropical and subtropical regions mainly in Asia and Americas [3, 4]. Estimates account for 390 million dengue infections per year of which 96 million present clinical manifestations [5]. The disease is endemic in 100 countries and approximately 40% of the world population is at risk of dengue infection [1]. By the end of the 20^th^ century, Brazil became the country with the highest notification rate, accounting for 98.5% of the American continent’s reported cases and the highest fatality rate in the sub region [1]. Since 2008, all four dengue serotypes (DENV-1 to DENV-4) can be found in the country [6].

NS1 Ag is a dengue highly conserved specific soluble glycoprotein essential for viral replication and viability that can be detected in dengue patients from day 1 up to 18 days after fever onset [7] with peak sensitivity in the first 3 days of fever onset [8]. It induces a strong humoral response and is known as a complement-fixing antigen [9].

Previous studies have evaluated the usefulness and the accuracy NS1 tests [10–14]. According to da Costa et al. the main factors influencing the diagnostic accuracy are the type of infection (primary versus secondary), viral serotype, geographical origins of samples, and the timing of sample collection [12].

Low NS1 test sensitivity in secondary dengue infections may be attributed to high levels of IgG in anamnestic response with immune complex formation impairing antigen detection by ELISA [9, 15]. Some studies have also noticed lower NS1 sensitivity in DENV-4 compared to other serotypes, in both primary and secondary dengue [12, 13]. Furthermore, immune complex dissociation techniques have proven to be important for the early diagnosis of HIV infections and most recently DENV [9, 16]. A study conducted in Rio de Janeiro with DENV-4 cases analyzed acid treatment/neutralization and heat dissociation, with a better performance for the latter [9]. In the present study, we compared the sensitivity of NS1 ELISA in heat dissociated and non-dissociated samples from acute DENV-4 patients.

## Methods

This study is part of a prospective cross-sectional pragmatic diagnostic study of clinical and diagnostic algorithms for dengue diagnosis conducted at Rio de Janeiro, Brazil during the 2013 dengue epidemic season (March and April). We used the Standards for Reporting of Diagnostic Accuracy Study (STARD) Guideline [17]. Eligible adult patients who presented to a public outpatient unit (Unidade de Pronto Atendimento-UPA 24H) within 72 h of a febrile illness without an evident source of infection were interviewed and examined by the investigators. At the time the study was carried out there were no evidences that other Flaviviruses, such as Zika were circulating in Rio de Janeiro [18].

Blood samples were collected and sera stored at -70 °C until the tests. The index test (Platelia^™^ Dengue NS1 Ag-ELISA), specific IgG and confirmatory RT-PCR were all performed at the Flavivirus Regional Reference Laboratory, IOC, FIOCRUZ.

The Platelia^™^ Dengue NS1 Ag-ELISA (Bio-Rad Laboratories, Marnes-La-Coquette, France) was performed in all samples according to the manufacturer’s instructions and described elsewhere, briefly the test is based on a one-step sandwich format microplate enzyme immunoassay to detect DENV NS1 antigen in human serum [10].

The same samples were further submitted to the heat dissociation method when 50 μL of the serum was added to 100 μL of RNA/DNAse free water and heated in a boiling water bath for five minutes, as described previously [9].

We defined as secondary cases those patients who presented positive IgG results within 72 h onset of disease [19]. The Dengue Virus IgG DxSelect^™^ ELISA (Focus Diagnostics, California, USA) was performed according to the manufacturer’s instructions in all samples to diagnose secondary dengue cases.

The viral RNA detection by RT-PCR was performed according to the technique described elsewhere [20]. Laboratory personnel were blinded to other laboratory data such as Platelia^™^ Dengue NS1 Ag-ELISA, IgG and RT-PCR when executing a specific test. DENV infections by other serotypes were excluded. Exploratory analysis was performed using SPSS^©^ v 17.0 (SPSS Inc., Chicago, Illinois). The MedCalc^©^ 14.8.1 program was used to calculate 95% confidence intervals (CI) for the Platelia^™^ Dengue NS1 Ag-ELISA sensitivity.

## Results

A total of 372 ambulatory patients were enrolled, with 10 exclusions (six presenting symptoms for more than 3 days and four due to infections to other DENV serotypes. The remaining 362 patients had their clinical profile previously described [21]. Briefly, 55.6% were female, the median age was 33 years old (ranging from 18 to 83) and the median time from the onset of illness was two days.

**Fig. 1:**
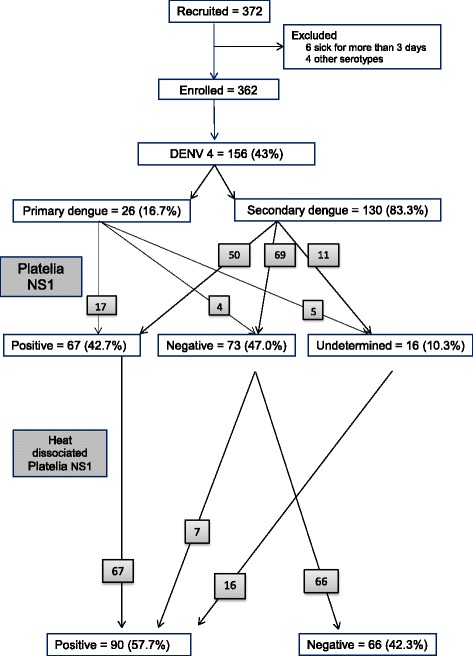
STARD [17] diagram DENV-4 patients in Rio de Janeiro, 2013

The RT-PCR identified DENV-4 in 42.8% (156/362) of the cases. From those, 16.7% (26/156) were classified as primary infection cases and 83.3% (129/156), as secondary ones, Fig. 1.

The NS1 ELISA performance as described by the manufacturer’s protocol in the 156 DENV-4 cases resulted in 43.0% (67/156) of positive cases, 46.8% (73/156) negative and 10.2% (16/156) indeterminate (Table 1). After the heat dissociation process, all 67 DENV-4 positive cases remained positive; 7 negative all 16 indeterminate cases became positive, as also described by Lima et al [9]. These results could jeopardize the theory of IgG immune complex formation; however the increase in sensitivity in those cases might occur due to an increased detection of monomeric NS1 forms [22].

**Table 1:** DENV-4 NS1 performance according to type of infection and method, Rio de Janeiro, 2013

Platelia™ Dengue NS1 Ag-ELISA	Results	Type of infection		Total *N* = 156
		Primary *N* = 26	Secondary *N* = 130	
Non-dissociated	Positive	17 (65.4%)	50 (38.4%)	67 (43.0%)
	Negative	4 (15.3%)	69 (53.3%)	73 (46.8%)
	Indeterminate	5 (19.2%)	11 (8.3%)	16 (10.2%)
Heat dissociated	Positive	22 (84.6%)	68 (52.3%)	90 (57.7%)
	Negative	4 (15.4%)	62 (47.7%)	65 (42.3%)

## Discussion

In our study, the overall sensitivity of the NS1 test in DENV-4 cases was 47.8%, quite similar to the 46.6% obtained by Lima [9], but lower than the 58% sensitivity obtained in da Costa’s meta-analysis [12], Table 2. This low overall sensitivity may be caused by the South American virus polymorphism lower overall viremia and lower NS1 secretion [12, 13]. The differences in the sensitivities in primary and secondary infections in our case before heat dissociation 80.9% vs 42.0% were also found in da Costa’s meta-analysis (94.6% vs 66%) [12]. Even attenuating the influence of secondary infections by heat dissociation, the sensitivities obtained were still low.

**Table 2:** Sensitivity of Platelia™ Dengue NS1 Ag-ELISA in acute DENV-4, Rio de Janeiro, 2013

	Platelia™ Dengue NS1 Ag-ELISA		Sensitivity (95% CI)	*p*-value
Overall sensitivity	Non-dissociated		47.8 (39.3 – 56.4)	*p* < 0.001
	Heat dissociated		57.7 (49.5 – 65.5)	
Type of infection	Primary	Non-dissociated	80.9 (58.1 – 94.6)	*p* < 0.001
		Heat dissociated	84.6 (65.1 – 95.6)	
	Secondary	Non-dissociated	42.0 (33.0 – 51.4)	*p* < 0.001
		Heat dissociated	52.3 (43.3 – 61.1)	

We demonstrated significant sensitivity increases after dissociation in both types of DENV-4 infections in agreement with other studies that used unspecific dengue serotype acid dissociated NS1 samples [15, 23].

This study has advantages since it was prospectively executed, the samples were clinically and laboratorially well characterized, and collected during a single outbreak in one outpatient unit increasing the chance of genotypic similarity, quite different from the previous study which was carried out during a 17 month period when genotypic heterogeneity is possible [9]. We were able to demonstrate the advantages of incorporating heat dissociation to the Platelia^™^ Dengue NS1 Ag-ELISA kit in order to increase its sensitivity, mostly in settings where circulating DENV-4 can be an issue. The study limitation was the lack of heterogeneity of viral strains that impairs the generalization of the results in other epidemic settings.

## Conclusions

Although time expending and the requirement of specific laboratory equipment the NS1 ELISA combined to heat dissociation could be a slightly better alternative for accurate dengue diagnosis.

## Abbreviations

CI: Confidence interval
CNPq: Brazilian National Council of Scientific and Technological Development
DENV-1: Dengue virus serotype 1
DENV-4: Dengue virus serotype 4
DNA: Deoxyribonucleic acid
ELISA: Enzyme-linked immunosorbent assay
FIOCRUZ: Oswaldo Cruz Foundation
IgG: Immunoglobulin G
IOC: Instituto Oswaldo Cruz
NS1: Nonstructural protein-1
RNA: Ribonucleic acid
RT-PCR: Reverse transcriptase-polymerase chain reaction.
STARD: Standards for Reporting of Diagnostic Accuracy Study
UPA 24H: Unidade de Pronto Atendimento
